# Methodological Considerations for Studies in Sport and Exercise Science with Women as Participants Part II: Guidance for Applied Studies in Elite Female Athletes

**DOI:** 10.1007/s40279-026-02442-3

**Published:** 2026-05-10

**Authors:** Kirsty J. Elliott-Sale, Richard J. Burden, Kathryn E. Ackerman, Louise M. Burke, Naama Constantini, Anthony C. Hackney, Rachel Harris, Franco M. Impellizzeri, Xanne A. K. Janse de Jonge, Constance M. Lebrun, Clare Minahan, Sophia Nimphius, Stuart M. Phillips, Sarianna Sipilä, Juliette A. Strauss, Paul Swinton, Craig Sale

**Affiliations:** 1 Department of Sport and Exercise Sciences, Manchester Metropolitan University Institute of Sport, Manchester, M1 7EL UK; 2 United Kingdom Sports Institute, Manchester, UK; 3 Women’s Health, Sports & Performance Institute, Boston, USA; 4 Mary MacKillop Institute for Health Research, Australian Catholic University, Melbourne, VIC Australia; 5 Shaare Zedek Medical Centre, The Hebrew University, Jerusalem, Israel; 6 Department of Exercise and Sport Science, University of North Carolina, Chapel Hill, NC USA; 7 Perth Orthopaedics and Sports Medicine Research Institute, Perth, WA Australia; 8 School of Sport, Exercise and Rehabilitation, Faculty of Health, University of Technology Sydney, Sydney, NSW Australia; 9 Griffith Sports Science, Griffith University, Gold Coast, QLD Australia; 10 Department of Family Medicine, University of Alberta, Edmonton, AB Canada; 11 School of Medical and Health Sciences, Edith Cowan University, Joondalup, Australia; 12 Department of Kinesiology, McMaster University, Hamilton, ON Canada; 13 Faculty of Sport and Health Sciences, University of Jyväskylä, Jyväskylä, Finland; 14 Research Institute for Sport and Exercise Sciences, Liverpool John Moores University, Liverpool, UK; 15 School of Health Sciences, Robert Gordon University, Aberdeen, UK

## Abstract

The purpose of this statement is to guide the design and conduct of high-quality sport and exercise science studies investigating the effects of varied ovarian hormone profiles on the health and athletic performance of elite female athletes. Beyond the menstrual cycle, these athletes present with a wide range of ovarian hormone profiles, including those associated with puberty and menarche, menstrual dysfunction, hormonal contraceptive use, pregnancy and postpartum, perimenopause, menopause and post-menopause. To effectively integrate knowledge of ovarian hormone profiles into high-performance sport and address the diverse needs of female athletes, future research and educational initiatives must encompass this broader spectrum of ovarian hormone profiles. However, to produce this knowledge, improvements in research design and conduct are necessary and can be facilitated by adopting reporting checklists as a structured approach to enhance transparency and reproducibility. We recommend that such quality assurance measures be integrated at the study design phase, along with clear research questions, appropriate study designs, and valid analysis methods. Consequently, the proposed guidelines aim to support researchers working in applied settings with elite female athletes in generating more robust and impactful evidence.

## Key Points

**Table:** 

Female athletes exhibit substantial inter-individual and intra-individual variability in ovarian hormone profiles; effective integration of this variability into high-performance sport requires structured health and performance frameworks rather than a one-size-fits-all approach.
Approaches to ovarian hormone tracking should be purpose driven, with methodological requirements differing between controlled research settings and applied practice.
Progress in women’s sport will remain constrained unless female athlete research consistently adopts rigorous female-specific methodological standards that adequately account for ovarian hormone status and variability.

## Introduction

### Background to the Statement

In 2021, Elliott-Sale et al. [1] provided guidance on selected methodological considerations for research in sport and exercise science (SES) with female participants, with the intention of raising research quality and advancing knowledge and practice. In addition to reviewing the state of the art and continued gaps in the field, they consolidated the experiences of veteran researchers and addressed some of the low-quality approaches often employed in this area. Here, we intend to address other methodological considerations related to SES studies using females as participants, with a specific focus on research in elite sport settings.

Over the Tokyo 2020 and Paris 2024 Olympiads, there has been growing interest in the influence of ovarian hormones on the health and athletic performance of elite female athletes, driven by the goal to increase medal tallies. Simultaneously, the development of professional women’s sport has also accelerated interest in ovarian hormones beyond their role in reproduction. For example, data from PubMed (accessed January 2026) show that publications relating to the menstrual cycle and sports performance rose from 13 in 2015 (i.e*.* start of the Tokyo Olympiad), to 45 in 2020, and reached 89 at the end of 2025 (i.e. following the Paris Olympiad). To date, these potential effects have not been definitively confirmed or refuted, despite an increase in the number of research studies. This means that there is currently no consensus amongst the scientific community on the existence, direction or magnitude of effects of ovarian hormones on elite women’s sports performance. The rise in quantity has regrettably outpaced an improvement in quality [2–4], resulting in mixed outcomes with no, limited or sometimes misleading applications for elite female athletes. This has created a need to increase not only the volume but also the quality of SES research involving female participants, such that it can be used, with a high degree of confidence, to inform and underpin the practices of elite female athletes.

Female athletes train, compete and recover through a variety of ovarian hormone milieus. These include transitional events such as puberty and perimenopause; phasal changes in endogenous oestrogens and progesterone (e.g*.* across the menstrual cycle); exposure to exogenous oestrogens and progestins (e.g*.* hormonal contraception users and hormone replacement users); and hormonal deprivation (i.e*.* menopause) [5–10]. These female-specific physiological states warrant proper investigation and consideration in the applied setting. Previous research activities and societal attention have primarily focused on the hormonal milieu associated with menstruation and the menstrual cycle. For example, despite being published within hours of each other and by the same authorship team, a review on the effects of the menstrual cycle on athletic performance [2] has been accessed 166 k times and cited 620 times in comparison to a review on the effects of oral contraceptives on athletic performance [3], which has been accessed 48 k times and cited 256 times. These metrics show a bias towards the menstrual cycle, despite up to half of elite female athletes reporting hormonal contraceptive use [5]. To truly serve the majority of elite female athletes, future research and educational strategies must include a wider selection of ovarian hormone profiles.

Therefore, despite growing female participation in Olympic and professional sports and increased research and societal focus on active women, high-quality data capturing the full spectrum of ovarian hormone profiles in elite female athletes remain scarce. Advancing research in this area requires rigorous and credible methodological frameworks.

### Approach to the Statement

Similar to the approach taken by Elliott-Sale and colleagues in 2021 [1], we have compiled methodological considerations based on previous research and expert knowledge, with a combination of primary sources and content specifically developed by the authors for this statement. We have triangulated published data with expert opinion and the experience of long-serving specialist researchers and practitioners, to strengthen the validity and credibility of our considerations. This approach works by comparing perspectives for convergence, complementarity or contradiction, rather than relying solely on a single perspective from previously published works, which is currently lacking in quality and quantity. In order to align with our focus on high-performance athletes, we have extended the authorship to include SES practitioners.

In principle, recommendations for the health and athletic performance of elite female athletes should be underpinned by insights gained from high-quality laboratory and applied investigations, with fundamental research supporting causal hypotheses about mediating mechanisms. However, in a research area where high-quality experimental evidence is still emerging, it is incumbent upon us to produce guidance derived from published empirical evidence and areas where guidance is informed by expert consensus and extensive domain-specific research and applied experience. Our intention is not to substitute opinion for evidence, but to provide structured methodological guidance in a developing research area, while clearly signalling the strength and source of the underpinning rationale.

### Scope of the Statement

The considerations described in this statement relate to ovarian hormone profiles and are intended for use in cisgender women (i.e. recorded female at birth with current identity aligned to this) and non-binary or gender diverse people who were recorded female at birth. We acknowledge the existence of other considerations involved in the inclusion of women participants in SES research, but these are outside the scope of this paper.

Numerous ovarian hormone profiles are recognised in this statement. In particular, the content is focused on menstrual cycle and associated irregularities as well as the use of hormonal contraceptives, reflecting that these ovarian hormone profiles currently represent the majority of elite female athletes [1]. It is also important to consider pregnancy, postpartum and menopause as more elite female athletes are beginning to incorporate pregnancy into their athletic careers [11, 12] and train and compete longer [13]. Wherever possible, we have included guidance for these populations; however, we acknowledge that this guidance is currently limited by the rare occurrence of pregnancy and menopause in elite sport, which has hindered the collection of observational or experimental data and restricted the guidance we can provide based on first-hand experience of these ovarian hormone profiles in high-performance sport. Acknowledging the importance of exercise and sport across the range of physical capabilities, this paper focuses on elite athletic populations as defined by McKay et al. [14] and specifically Tier 4 and 5 athletes at the elite/international and world class levels.

While this paper is focused on the applied setting, some of the content will also apply to laboratory studies. To avoid content duplication, this statement should be considered alongside other methodology-focused publications [15–19]. All of these publications [15–19] include members of the current authorship team and are therefore aligned with the content present herein. It should be noted that other relevant resources exist, which do not include any of the current authors, including [20–22]. We signpost readers to these resources for other perspectives. Whilst some overlap exists between these publications [15–22] and the present statement, herein we have prioritised novel content, whilst also consolidating some previously described concepts, to provide readers with a comprehensive and expansive set of considerations. This statement is focused explicitly on research in elite female athletes, where methodological constraints, performance environments, and athlete management considerations differ substantially from those in general or recreational populations. Within this statement we have integrated ovarian hormone profile considerations, elite sport performance demands, and real-world applied research constraints into a single structured framework designed specifically for studies conducted in high-performance sport settings.

It is outside of the scope of this paper to summarise or critique individual research studies involving ovarian hormones and elite female athletes. We have presented our main methodological concerns about assumed ovarian hormone profiles elsewhere [2–4]. This statement represents the pertinent learning from previous studies, without providing specific study details.

### Purpose of the Statement

The purpose of this statement is to guide the design and conduct of high-quality SES studies that investigate whether the various ovarian hormone profiles experienced by elite female athletes have a meaningful *effect* on their health and athletic performance. Such research should produce methodologically robust findings in order to inform solutions to the scenarios faced specifically by elite female athletes in relation to their reproductive hormonal milieu.

## Considerations

### Definition of Terms

We have provided a list of key operational terms in Table 1, which should be used in combination with those provided by Elliott-Sale et al. [1]. The *operational* terms are taken from the Oxford Language and Medical Dictionaries and are included to avoid any ambiguity with the terms used herein. The *new* and *updated* terms have been produced specifically for this statement and are intended to result in more precise participant classification and selection (i.e*.* inclusion/exclusion criteria) than previously employed. It should be noted that alternative definitions are available and permitted, but these should be described, and when necessary, justified, when used in future studies and texts. The purpose of providing definitions is to remove ambiguity, prevent misinterpretation, and allow for meaningful comparisons between studies/texts. Additionally, we encourage future studies to adopt the updated terminology for abnormal uterine bleeding, as outlined by Oleka et al. [23], as well as the staging system for reproductive age by Harlow et al. [24] when describing the current state of female athletes in relation to the hypothalamic-pituitary-ovarian function that occurs before and after the final menstrual period.

**Table 1:** Hormonal and operational terms used within this statement and intended for use in future studies in sport and exercise science with elite female athletes as participants

Hormonal terms	Defined as
Progestogens	Natural or synthetic progestational steroid hormones
Progesterone	Natural steroid hormone, which plays an important role in pregnancy and other non-reproductive functions
Progestin	Synthetic progestogen
Oestrogens	Primary female sex hormones, which play important roles in both reproductive and non-reproductive systems
17-Beta oestradiol	Primary form of oestrogen during reproductive years and the most potent form of oestrogen
Oestrone	Primary form of oestrogen after menopause
Oestriol	Primary form of oestrogen during pregnancy

Operational terms	Defined as
Assumption	A thing that is accepted as true or as certain to happen, without proof
Verification	The act of verifying something (proving or checking that it exists, or is true or correct)
Objective	Not influenced by personal feelings or opinions in considering and representing facts
Subjective	Based on or influenced by personal feelings, tastes or opinions
Perceive	Interpret or regard (someone or something) in a particular way
Lived experience	A depiction of a person’s experiences and decisions, as well as the knowledge gained from these experiences and choices
Opinion	A view or judgement formed about something, not necessarily based on fact or knowledge
Self-report	An approach requiring participants to respond to questions without interference; can be valuable in obtaining perspectives, views and opinions, but subject to bias and limitations (e.g. lack of knowledge)
Symptom	An indication of a disease or disorder noticed by the patient (i.e*.* subjective evidence)
Sign	An indication of a particular disorder that is detected by a physician while examining a patient (i.e. objective evidence)
Cyclicity	The quality or state of something that occurs or moves in cycles: a cyclic quality or state

Updated terms from Elliott-Sale et al. [1]	Defined as
Naturally menstruating (abbreviation: NM)	Women who experience regular menstruation, with menstrual cycle lengths ≥ 21 days and ≤ 35 days
Naturally menstruating with confirmed luteinising hormone surge (abbreviation: NM-LH)	Women who experience regular menstruation, with menstrual cycle lengths ≥ 21 days and ≤ 35 days, and have evidence of a surge in luteinising hormone (LH) **Rationale:** A surge in LH usually means that ovulation is about to occur; however, it is possible to experience an LH surge that is not sufficient to trigger ovulation. One of the main reasons for using the LH surge, outside of its link to ovulation, is to facilitate the timing of the Phase 4 blood sample for the determination of progesterone
Eumenorrheic-progesterone only (abbreviation: EUM-P)	Women who experience regular menstruation, with menstrual cycle lengths ≥ 21 days and ≤ 35 days, have evidence of a surge in LH, and have a Phase 4 progesterone concentration > 16 nmol∙L^−1^
Eumenorrheic (abbreviation: EUM)	Women who experience menstruation regularly, with menstrual cycle lengths ≥ 21 days and ≤ 35 days resulting in nine or more consecutive menstrual periods per year, have evidence of a surge in LH, have a hormonal profile for each phase within defined ranges and have not used hormonal contraception for 3 or more months prior to recruitment **Defined ranges:** Phase 1 is characterised by low levels of oestradiol and progesterone; Phase 2 is characterised by the highest concentration of oestradiol (higher than Phase 1, 3 and 4) and low progesterone levels (< 6.4 nmol∙L^−1^); Phase 3 oestradiol is higher than Phase 1 but lower than Phase 2 and 4 and progesterone is lower than 6.4 nmol∙L^−1^; Phase 4 oestradiol is higher than Phase 1 and 3 but lower than Phase 2 and progesterone is greater than 16 nmol∙L^−1^
Eumenorrheic + (abbreviation: EUM +)	Women who experience menstruation regularly, with menstrual cycle lengths ≥ 21 days and ≤ 35 days resulting in nine or more consecutive menstrual periods per year, have evidence of ovulation via transvaginal ultrasonography, have a hormonal profile for each phase within defined ranges and have not used hormonal contraception for 3 or more months prior to recruitment

Clarified terms from [1]	Defined as
Phase 1, 2, 3 and 4	The phases of the menstrual cycle described in Elliott-Sale et al. [1] are based on significant changes in the concentration of oestradiol and progesterone across the menstrual cycle. In most research studies, observations are made in Phase 1 and 4, or in Phase 1, 2 and 4. Phase 2 can be difficult to predict and requires retrospective confirmation via blood samples, which may result in the exclusion of data that do not fit in the defined range. Phase 2 does, however, provide the phase with the peak in oestradiol levels. Although rarely used in research studies, Phase 3 is easier than Phase 2 to predict and rarely results in the exclusion of data that do not fit in the defined range. If Phase 3, rather than Phase 2, is used, the phase with the highest oestradiol is not assessed

New terms	Defined as
Ovarian hormone profiles	An umbrella term used to describe all hormonal profiles associated with cyclical or phasal fluctuations (with the exception of diurnal, nocturnal, or circadian variation) in oestrogens and progesterone experienced by women across the lifespan **Rationale:** In sport settings (and its associated media), the “*menstrual cycle”* has sometimes been used to describe all situations associated with reproductive physiology in female individuals. This is a misnomer that can limit the depth and breadth of research and its potential application and educational potential in this field. The term *ovarian hormone profiles* is intended to broaden this research area, thus expanding the translation factor of future research to more female athletes and highlighting the multiplicity of ovarian hormone profiles experienced by female individuals across the lifespan. Researchers and other end users should be aware that the menstrual cycle is just one of the ovarian hormone profiles that female individuals can experience during their lifetime
Phase A, B and C	These terms are used to describe distinct phases of the menstrual cycle based on symptomology. Specifically, Phase A refers to the days during menstruation [typically lasting 2–7 days]; Phase B refers to the time just before and during ovulation [typically lasting 24–48 h]; and Phase C refers to the days [usually 7 days but up to a maximum of 14 days] prior to menstruation (Fig. 1)

### Research Question

This statement focuses on the reproductive and non-reproductive effects of ovarian hormones in elite female athletes. While the reproductive and health effects of oestrogen and progesterone are well known, their non-reproductive effects on the physiological determinants of athletic performance or responses to exercise are not fully understood. To this end, the underlying mechanisms behind such effects should be considered and clearly stated. This requires a priori thinking to develop a solid rationale for the research question. This should not be a post hoc approach when examining data in an attempt to reverse engineer a finding, which is only acceptable for hypothesis generation. Moreover, studies should not be conducted solely on the basis of novelty (i.e. not examined in female individuals before) or on account of prior published data indicating a potential ovarian hormone impact on a particular exercise-related outcome. Whilst the latter can provide a useful justification to pursue a research question in this area, the quality of such evidence needs to be established prior to using it to underpin more research; meaning that research with more rigorous methodologies may need to be conducted to verify or refute earlier findings.

The ways in which ovarian hormones may exert their effect in female athletes are six-fold; (i) linked to the act of bleeding and its management; (ii) associated with cultural and psychosocial responses, including stigma and shame; (iii) caused by associated signs and symptoms; (iv) via ill-health; (v) informed by perception; and (iv) through a physiological pathway. Studies should explicitly state which effect(s) they are investigating in their research question and should employ a variety of research methods, both qualitative and quantitative, to address different research questions.

#### Bleeding, Hygiene Management and Practical Considerations

The act of bleeding itself can create logistical challenges for female athletes. Hygiene management requires constant vigilance, including access to sanitary products, appropriate clothing, and adequate facilities during training and competition. In some contexts, the availability of sanitary products or opportunities to change them may be limited, increasing stress and disrupting performance preparation [25]. Even when products are accessible, athletes often report that they can be uncomfortable, inconvenient, or perceived as incompatible with certain types of sport apparel [26]. These practical demands can distract athletes, necessitate adjustments to routines, and interfere with optimal performance. Similar challenges may be experienced by athletes using hormonal contraceptives, who may experience irregular bleeding, and by perimenopausal athletes, who often face unpredictable bleeding patterns.

#### Cultural and Psychosocial Influences

Beyond the practical aspects of managing menstruation, cultural and psychosocial factors exert an additional layer of influence. Stigma surrounding menstruation persists, with many athletes feeling pressure to conceal their menstrual status and avoid disclosing menstrual difficulties [27, 28] in certain sporting cultures, where open discussion of menstrual health is limited or considered inappropriate, these pressures may be intensified. Athletes can experience feelings of embarrassment, shame or fear of judgement, which increase their mental load during training and competition. Such cultural silences may also be reinforced by the broader sporting environment, including the absence of structured support for menstrual health within teams or limited acknowledgement of menstruation by coaches and staff. These psychosocial influences can affect confidence, concentration and willingness to engage fully in sport. Together, they highlight how menstruation is not only a physiological process but also a culturally mediated experience that can shape performance outcomes.

#### Symptoms

**Fig. 1:**
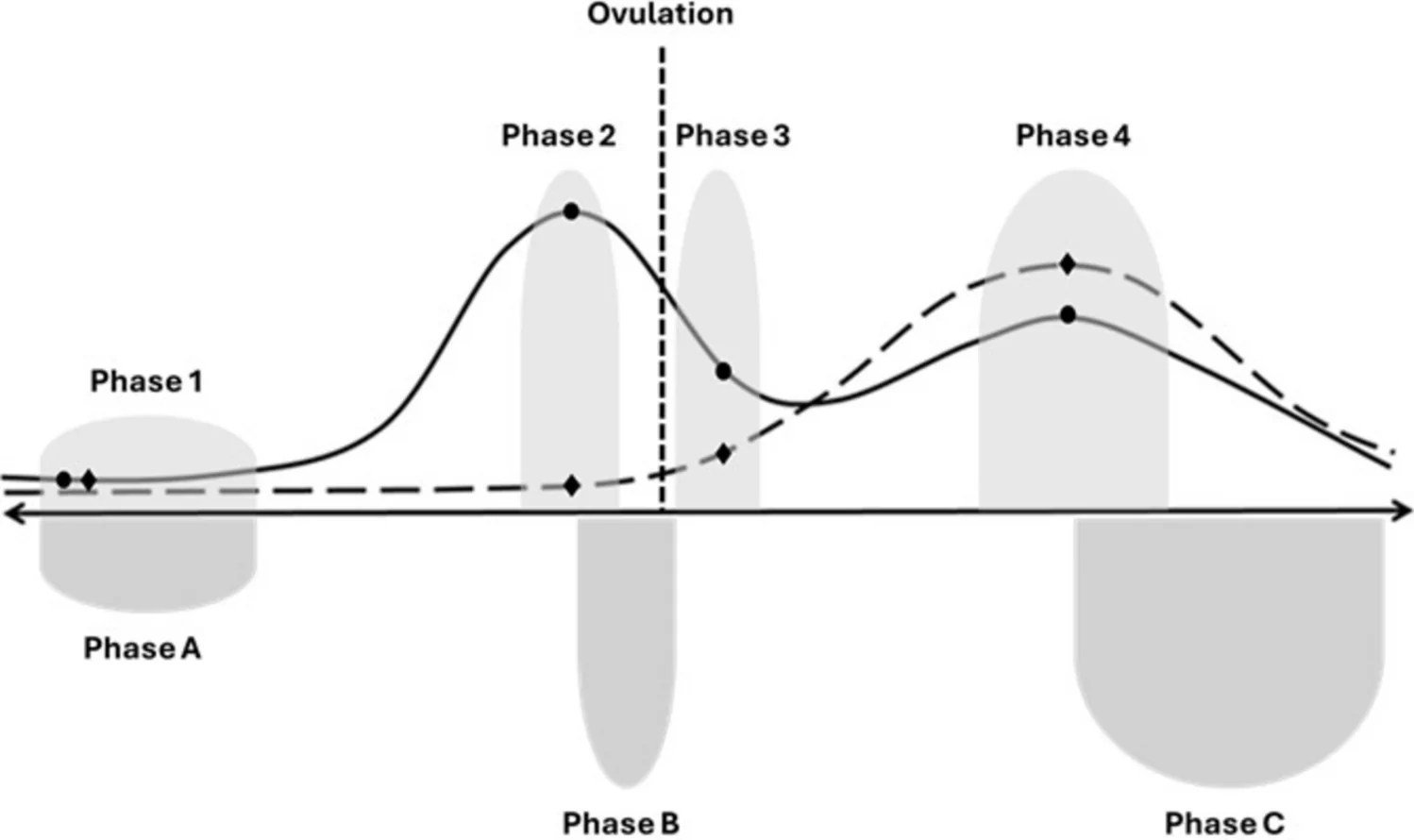
Phases of the menstrual cycle based on significant changes in ovarian hormone concentration (Phases 1–4) and the occurrence of symptoms (Phase A-C). Phase A refers to the symptoms associated with dysmenorrhea, Phase B with mittelschmerz and Phase C with premenstrual syndrome and premenstrual dysphoric disorder

The symptomology associated with the menstrual cycle is nuanced. There has been a predisposition to attribute all adversity, either physical or emotional, experienced by female athletes to their menstrual cycle. Studies [5, 8, 10] reveal that many female athletes report symptoms such as abdominal pain, mood swings and fatigue throughout various parts of the cycle rather than in a single phase. Whilst the majority of symptoms are typically recorded in the premenstrual (Phase C) and menstrual (Phase A) phases, symptoms have been registered across all days of the cycle; meaning that they may not always correspond precisely with ovarian hormone changes (Fig. 1). As such, there is a need to establish which symptoms are attributable to the menstrual cycle (i.e. are cyclical) and those that have other causes. Long-term monitoring (e.g. three or more consecutive cycles) should be used to examine symptomology patterns. Symptoms that consistently occur in line with particular phases of the menstrual cycle should be identified and suitable interventions initiated. Sporadic symptoms should be investigated further, with a view to establishing their cause and implementing appropriate interventions.

Symptom tracking protocols should avoid ‘leading’ the athlete (i.e. asking questions that suggest the desired answer or put words in the athlete’s mouth). Ideally, athletes should report their symptoms and severity in real time (i.e. as opposed to at a set time each day, e.g. daily monitoring first thing in the morning). Real-time reporting refers to reporting the symptom as soon as possible after its onset. Examples of how this can be facilitated include online forms, which can be accessed on mobile devices, or via QR codes posted around training and competition facilities. The perceived impact of the symptom should also be noted and checked against the actual effect if possible. Effective intervention programmes should then be designed, implemented and evaluated in response to any cyclical symptoms, which are aligned with particular phases of the menstrual cycle or other ovarian hormone profiles (e.g. perimenopause). Interventions can be based on:

(i) Accommodating adverse symptoms; this practice is known as autoregulation and should not be referred to as menstrual-cycle phase-based training. Autoregulation is a training framework that was established in the 1940s (for a comprehensive review, see Greig et al. [29]) and relates to the manipulation of training based on actual performance or perceived capability to perform. Autoregulation is not exclusive to female athletes and is commonly used by male athletes.
(ii) Mitigating adverse symptoms such that athletes are able to train and compete to the best of their ability on any given day of the menstrual cycle.

Either strategy is acceptable; however, the design and implementation of effective mitigation interventions should be prioritised if an athlete wishes to maximise their training potential, and health and well-being. A proactive approach is preferable over a reactive approach.

It is also important to note that female athletes can experience positive effects during the menstrual cycle; for example, some elite female athletes have reported feeling stronger during certain phases of their cycle (findings from 320 top-flight European female football players) [30], and Paula Radcliffe is believed to have broken the world-record marathon time in 2002 while menstruating. Indeed, many studies have shown female athletes prefer to perform at all phases of their cycle, again mirroring the nuanced individual lived experiences [31].

Several studies [5, 8, 10, 32, 33] have asked female athletes to report their first-hand experiences of using hormonal contraceptives. Therein, many female athletes have reported symptoms, which they attributed to their hormonal contraceptive use. With the exception of some hormonal coil users (i.e., intra-uterine system), who often experience a typical menstrual cycle hormonal profile, the remainder of hormonal contraceptive users experience downregulation of their endogenous ovarian hormones and a synchronous exogenous hormonal profile, containing an oestrogen and progestin or progestin only. As such, they do not experience the four phases of the menstrual cycle with significantly different ovarian hormone concentrations (i.e. Phases 1–4) or the three phases of the menstrual cycle based on symptomology (*i.e.*, Phases A, B and C). When negative symptoms are reported, effective mitigation strategies should be designed, implemented and evaluated, by a multidisciplinary team with expertise in gynaecological endocrinology and exercise physiology, to limit any effects on training or competition performance, starting with consideration of an alternative type of hormonal contraceptive with fewer side effects.

Perimenopause is characterised by wide hormonal fluctuations and menstrual irregularities that have been associated with a higher risk of symptoms experienced throughout the body. Vasomotor (e.g. sweating and hot flashes), psychological (e.g. mood and sleep disturbances) and genitourinary (vaginal and urinary tract complaints) symptoms are typically reported by many women at this stage [34]. The beginning of the transition phase is gradual and the timing and symptomology are highly individual. Therefore, many female athletes may experience symptoms that temporarily change their health and quality of life without linking these to perimenopausal hormonal changes. At this stage, women need support and understanding to manage their symptoms. If the link between hormonal changes and symptomology is evident and symptoms are bothersome, hormonal replacement therapy is an effective treatment option [35]. In studies, this phase of women’s reproductive ageing may be difficult to assess in real-time because of the hormonal fluctuation, variable length of menstrual cycles, and transient symptoms. Earlier studies have, however, made considerable effort to characterise their female participants into different phases of hormonal ageing [36, 37]. While perimenopause is rarely experienced by elite athletes, certain sports (e.g. shooting, archery, equestrian) and populations (e.g. paralympians) of elite athletes may have higher numbers of women experiencing perimenopause.

#### Ill Health

Because of the secondary effects of oestrogen and progesterone on several physiological systems, sub-optimal ovarian hormone profiles, such as those experienced during different types of menstrual dysfunction or associated with menopause, may lead to ill-health. For example, oligo-amenorrheic athletes or athletes with premature ovarian insufficiency or early menopause are likely to have poor bone accrual and low bone mineral density and be at higher risk of developing osteopenia and osteoporosis than those with oestrogen sufficiency (i.e. eumenorrheic athletes) [38, 39]. Deteriorations in bone health have been associated with an increased risk of bone injuries, for example Heikura et al. [40] showed that bone injuries were ∼4.5-fold more prevalent in amenorrheic athletes compared with their eumenorrheic counterparts. This example illustrates one way in which ovarian hormone-related ill-health has deleterious effects on a female athletes potential to train and compete.

#### Perception

The idiom ‘perception shapes reality’ is well known. Perception act as a lens through which experiences and information are filtered. In recent years, social media has transformed the once taboo topic of menstrual cycles and menstruation into a widely discussed subject. In 2025 Pfender et al. [41] conducted a quantitative content analysis of 100 TikTok videos on ‘cycle syncing’ (i.e. tailoring diet and training for each phase of the menstrual cycle). For context, at the time of study, the hashtag #cyclesyncing had received 285 million views, primarily from viewers aged 18–24 years. This study showed that only one third of creators provided credentials, almost none (4%) mentioned scientific evidence, some promoted a negative discourse about hormonal contraceptive use and others provided problematic messaging about women’s reproductive health. This example highlights the pervasive rise of pseudo-science on social media related to ovarian hormone profiles. As such, today’s female athletes are faced with navigating anecdotal rather than scientifically validated information promoted by content creators with a substantial lack of credibility. This means that, now more than ever, female athletes need trustworthy high-quality evidence to correctly shape their perceptions and prevent adverse effects on their health and performance through misconceptions.

#### Physiology

The physiology pathway that underpins the effects of oestrogen and progesterone relies on the amount of free hormone and the relative number of functional receptors. The total concentration of hormones circulating in the bloodstream is made up of free and bound fractions, with the free form interacting with its specific receptors found on target tissues throughout the body. Because some of the bound fraction can occasionally disassociate and exert an effect, the ‘bioavailable’ portion of the hormone is not a set proportion of the total concentration. The reproductive organs are the principal target tissues of the free portion of ovarian hormones and typically contain the greatest expression of the specific receptors (i.e. PR-A and PR-B for progesterone and ERα and ERβ for oestrogen). Yet many other organs and tissues express receptors for these hormones, although typically at far lower rates. For a hormone to affect its target cells, the cell must express adequate numbers of hormonal receptors, these receptors must be functional and have an appropriate affinity for the hormone, and the cell itself must have adequate post-receptor amplification mechanisms to respond (i.e. viable downstream events need to be available) [42]. Although the expected enhanced receptor expression in reproductive organs should support a more viable and robust uptake of the free portion of the ovarian hormones, specific evidence on how much non-reproductive tissue receptors uptake of the free hormonal portion is unclear [43]. The latter certainly occurs, but in the hierarchy of hormonal physiological regulations and the criticality of maintaining the species, one would expect there to be a reproductive predominance of hormonal utilisation. It makes sense that the higher the circulating free hormone concentration, the higher the potential for effects in other (i.e. non-reproductive) target tissues expressing the relevant and active hormonal receptors and the capability for post-receptor amplification. It is difficult to ascertain, however, the magnitude of effect of any specific ovarian hormone across multiple target tissues at any given point in time, as there are multiple other factors that might also influence this (e.g. metabolic state, age, hormonal interactions, diet and lifestyle factors). To date, the effects of fluctuations in endogenous oestradiol and progesterone, during puberty, across the menstrual cycle, throughout pregnancy and postpartum, and during the menopausal transition, on the determinants of athletic performance or the response to exercise training are inconclusive and require more research.

Hormonal contraceptives contain synthetic sex hormones (oestrogen and progestin), which are easily absorbed and are typically more potent than their endogenous counterparts [44, 45]. The synthetic ovarian hormones suppress the hypothalamic-pituitary-ovarian axis, preventing ovulation, which leads to lower levels of endogenous hormones. The dose–response relationship (i.e. the interaction between potency and mass action) is the same regardless of whether the ovarian hormones are endogenous or exogenous [46]. However, the exogenous hormones affect potency (i.e. the product of the affinity of the effector and its efficacy at the effector site because of the chemical structure) as well as the mass action (i.e. a function of affinity and concentration, which is affected by varying levels and/or half-life of the synthetic hormones), and therefore also need to be considered when studying elite female athletes. Similar to the menstrual cycle, the effects of exogenous sex hormones on athletic performance in elite female athletes remain unknown and warrant further consideration.

### Data Interpretation: Ensuring Alignment of Research Questions, Study Design and Analyses

Accurate quantification of ovarian hormone profiles is a necessary, but not sufficient, condition for producing high-quality research. Indeed, high-quality research requires a clearly defined scientific question, which must then be translated into a corresponding statistical question. Bias can be introduced at each step of the research process, including ambiguity in study aims that fail to identify appropriate estimands (i.e*.* the specific quantities researchers aim to estimate) [47–49]. A clearly specified estimand ensures alignment between study aims, data collection and analytical approach; a misalignment that is unfortunately common [50]. Accordingly, data analysis and interpretation should be guided by the estimand, which also reflects the study’s intent (e.g. confirmatory or exploratory). Simplifying for brevity, a tripartite operational model of research categorises the main goals into three types: description, prediction and explanation [51, 52]. Explanation often focuses on identifying causal relationships, a concept sometimes referred to as a causal explanation. In this article, we refer to the *effect* of ovarian hormone profiles on health and athletic performance, which implies a causal question.

Interpreting results from descriptive and predictive studies as causal is misleading and can result in inappropriate conclusions [53–55]. Descriptive studies, which summarise data or identify associations, are valuable for hypothesis generation but cannot establish or support causal relationships. These studies also cannot reliably inform recommendations unless the speculative nature of the interpretation is explicitly acknowledged. Similarly, most predictive studies, such as those using hormonal profiles as features in forecasting models, aim to optimise predictive accuracy rather than infer cause. In such cases, hormonal profiles may improve prediction but should not be interpreted as causal determinants. The latter requires evidence of causal mechanisms before any factor can be used as a target for intervention [51, 53, 56, 57]. This evidence should be built on cumulative knowledge and replications, in order to avoid the fallacy of single trials that place undue confidence in isolated findings that can lead to premature or unsupported changes in practice.

To establish causation, experimental studies such as randomised controlled trials are considered the gold standard. However, in the context of ovarian hormonal profiles, ethical and practical constraints often render such designs unfeasible. In such cases, well-designed observational studies can serve as viable alternatives for causal inference, provided key assumptions are met and transparently articulated. While the detailed methodology for observational causal inference is beyond the scope of this article, extensive guidance is available [58, 59]. Here, we emphasise the critical role of background knowledge and the confirmatory nature of causal research. Indeed, causal inference requires the explicit definition and transparent presentation of causal assumptions, often communicated in graphical or mathematical forms [60–62]. This process necessitates the conceptualisation and development of theories and hypotheses. In the context of ovarian hormonal profiles and their effects on athletic performance or health outcomes, this involves identifying potential delayed or immediate effects, induction time, latent periods and other relevant factors. This further highlights the complementarity of basic and laboratory (mechanistic) research with “applied”" research [63, 64].

To assess potential causal mechanisms linking changes in hormonal profiles, such as those across the menstrual cycle, to athletic performance outcomes, interdisciplinary teams are recommended. These teams are likely to require physiologists, clinicians, physical performance experts, statisticians, epidemiologists and data analysts to enable comprehensive study design, measurement and interpretation. Such collaboration supports the development of appropriate datasets and analytical models, which are critical for evaluating whether robust causal evidence can be generated. In contrast, much of the existing literature over-relies on associations that are misinterpreted as evidence of causation, often without employing the necessary methodological frameworks [54, 55, 65]. While such misinterpretations are known fallacies, they also reflect well-documented cognitive tendencies to perceive causality even when it is unwarranted [66]. Some of the authors of this article acknowledge having made similar interpretive errors in past work. However, it is time to progress and improve, learning from our past mistakes. To support accurate interpretation of research involving ovarian hormonal profiles and performance outcomes, appropriate designs (including correct sampling of participants) and analyses are just as important as the correct assessment of hormonal profiles. Collaborative and big-team science approaches offer a promising path forward, combining diverse expertise with larger and more representative datasets. This is particularly important in SES, where female participants have historically been under-represented, and where robust generalisable findings are urgently needed.

### Data Collection: Establishing Common Ovarian Hormonal Profiles

Establishing ovarian hormone profiles requires a two-step approach, using subjective (i.e. unsubstantiated) and objective (i.e. substantiated) measures; in lay terms, first you ask, then you confirm. Elliott-Sale et al. [17] recently developed a robust tool, using self-reported data, for classifying ovarian hormone profiles (step one) in post-menarcheal to perimenopausal female athletes. The resultant classification can be used to determine which verification process (step two) is needed. The objective verification of ovarian hormone profiles in elite female athletes (i.e. the second step) is now described.

#### Menstrual Cycle

Data from Allaway et al. [67] illustrate that unless menstrual cycle phases are verified (i.e. the fluctuations in endogenous oestradiol and progesterone across the menstrual cycle), subtle menstrual irregularities can go undetected. Their data show that, despite regular menstruation, female individuals can experience menstrual disturbances without any discernible signs. In the absence of objective ovarian hormone data, the *assumed* ovarian hormonal profile may not match the *actual* profile. A comprehensive review of the issues associated with using estimated and assumed menstrual cycle phases (i.e. without verification) is provided by Burden et al. [16] and Elliott-Sale et al. [4].

To date, there is no consensus on which measurements to use to verify menstrual cycle phases, although there is agreement on the need for both subjective and objective measures. We propose a spectrum-based approach (Table 2), depending on the research question or practical need (i.e. related to health and/or athletic performance) and the availability of resources (budget, availability of suitable qualified staff, time, equipment). Although we recommend the use of blood samples throughout these guidelines, factors such as cost and resources often necessitate the use of other objective measurements (e.g. other biological fluids such as saliva) provided these can provide accurate and sensitive data. Regardless of the specimen type, the timing and frequency of sampling, as well as the practical aspects of the analytical technique need to be considered and optimised.

**Table 2:** Establishing menstrual cycle phases in elite female athletes

Spectrum of burden	Resource[s]	Resource burden: cost	Approach	Athlete burden: time and effort	Health-related implication[s]	Performance-related implication[s]
Low	Calendar [paper or electronic]	No cost for free online calendar app or low cost for paper calendar	NM: Record when menstruation occurs	Negligible time/effort	Establishes NM and eliminates AUB-frequent, AUB-infrequent and amenorrhea	Allows for a comparison of outcomes on bleeding (Phase 1) vs non-bleeding days
#	Calendar + urinary ovulation tests	+ Cost for at home testing kits	NM-LH: record when menstruation occurs and check for LH surge	+ Required to test urine every day for 3–10 consecutive days (5-min daily task)	Establishes NM, eliminates AUB-frequent, AUB-infrequent and amenorrhea and significantly reduces the possibility of AUB-O	Allows for a comparison of outcomes between Phases 1, 2 and 3
^**#**^**This option [NM-LH] is advised for most elite female athletes who intend to use their data to inform their practice**						
*	Calendar + urinary ovulation tests + one-off blood sample/analysis	+ Cost for at home kit or blood draw plus laboratory service	EUM-P: record when menstruation occurs, check for LH surge, measure progesterone levels	+ Required to provide a blood sample either at home or at a suitable location necessitating travel, time and inconvenience	Establishes NM, eliminates AUB-frequent, AUB-infrequent and amenorrhea, significantly reduces the possibility of AUB-O and AUB-E	Allows for a comparison of outcomes between all phases (1–4)
^*^This option [EUM-P] is advised for elite female athletes who are taking part in applied research studies						
^	Calendar + urinary ovulation tests + repeated blood samples/analyses	+ Cost of multiple at home kits or blood draws plus laboratory service	EUM: Record when menstruation occurs, check for LH surge, and measure oestradiol and progesterone levels in each phase	+ Required to provide multiple blood samples either at home or at a suitable location necessitating travel, time and inconvenience	Establishes NM, eliminates AUB-frequent, AUB-infrequent and amenorrhea, and AUB-E and almost eliminates AUB-O	Allows for a comparison of outcomes between all phases (1–4), using a higher quality research design, resulting in a higher degree of certainty in the findings and interpretation of findings
**^This option [EUM] is advised for elite female athletes who are taking part in laboratory-based research studies**						
High^!^	Calendar + transvaginal ultrasound + repeated blood samples/analyses	+ Cost of sonography	EUM*: Record when menstruation occurs, undertake transvaginal ultrasonography, and measure oestradiol and progesterone levels in each phase	+ Required to travel to a suitable location for ultrasonography necessitating travel, time and inconvenience	Establishes NM, eliminates AUB-frequent, AUB-infrequent and amenorrhea, AUB-O and AUB-E	Allows for a comparison of outcomes between all phases (1–4), using the highest quality research design, resulting in a higher degree of certainty in the findings and interpretation of findings
^**!**^This option [EUM*] is advised for situations when absolute confirmation of ovulation is required as part of the research design or for health reasons						

*AUB* abnormal uterine bleeding, *AUB-E* luteal phase defect, *AUB-O* anovulation, *EUM* eumenorrheic, *EUM-P* eumenorrheic progesterone only, *LH* luteinising hormone, *NM* naturally menstruating, *NM-LH* naturally menstruating with confirmed LH surge

#### Hormonal Contraceptives

In the case of cyclical hormonal contraceptives, the active versus inactive phase (e.g. pill-taking vs pill-free days, patch-wearing vs non-wearing days, vaginal ring-wearing vs non-wearing days) should be noted, with measurement of the corresponding concentrations of both endogenous (i.e*.* to confirm downregulation) and exogenous (i.e*.* to determine dose–response) hormones. In the case of progestin-only contraceptives, the time since insertion (i.e. implant and intra-uterine system) or injection should be noted with measurement of the corresponding concentrations of both endogenous (i.e*.* to confirm downregulation) and exogenous (i.e. to determine dose–response) hormones. As a reminder, the concentration of exogenous progestin peaks in the immediate weeks (e.g. injection) and months (e.g. implant) and declines steadily throughout the remaining timeframe. As such, there is not an on/off phase per se, rather a high/lowering exogenous profile. For further reading on the pharmacokinetics of hormonal contraction see [68].

#### Menopause

During the last 20–30 years, there has been an increase in the number of master athletes participating in competitive sports [13]. Even though there is no single definition or age limit for master athletes, reproductive ageing and eventually menopause affect most of the elite female athletes participating in competitive sports at the master’s level. The Stage of Reproductive Aging Workshop + 10 (STRAW + 10) defined the critical changes to the hypothalamic-pituitary-ovarian axis, which occur during reproductive ageing and provided guidance on how to characterise these changes into stages [18]. As hormonal ageing is quite individual, it is especially important that classification of the stages of reproductive ageing (e.g. reproductive, menopausal transition, post-menopause) is not based on age alone. Applying the STRAW + 10 guidelines in SES research with female master athletes as participants is crucial for the verification of menopausal stage.

#### Pregnancy and Post-Partum

There are many early symptoms associated with pregnancy: the absence of menstruation (*i.e.*, secondary amenorrhea), fatigue, breast tenderness, heightened emotions and “morning sickness”. Pregnancy can be confirmed using a home pregnancy test, which detects human chorionic gonadotropin in urine, or via a blood sample or ultrasound performed by a clinician. Pregnancy is usually divided into trimesters: first trimester conception to 12 weeks; second trimester 13–27 weeks; and third trimester 28–40 weeks. Pregnancy causes chronic changes in the female reproductive environment, resulting in supraphysiological concentrations of endogenous oestrogen and progesterone (see Elliott-Sale [69] for a comprehensive overview of ovarian hormones during pregnancy). During pregnancy, the increase in 17-β-oestradiol and progesterone can be as much as 73-fold and nine-fold [70]. Following pregnancy, and in the absence of lactational amenorrhea or hormonal contraceptive use, oestradiol and progesterone return to menstrual cycle levels. The stage of pregnancy should be classified initially (i.e. first, second or third trimester) and then confirmed by measuring oestradiol and progesterone levels. Following parturition, the postpartum stage should be classified (i.e. weeks since birth—up to a maximum of 52 weeks, breastfeeding status, hormonal contraception use) and subsequently verified using either the menstrual cycle protocol or hormonal contraceptive protocol described above. In the case of lactational amenorrhea, the downregulation of oestradiol and progesterone can be confirmed using a blood sample analysis if required.

#### Blood, Saliva and Urine Measurements

Blood measurements of ovarian hormones are considered the gold standard. While urinary and salivary hormone measurements have been suggested as a more practical, non-invasive solution for serial ovarian hormone profiling (for a review, see Huang et al. [71]), there are important caveats to remember when using these biological specimens rather than blood. Such considerations include: (i) hormone values from saliva do not equate precisely with blood hormone levels as only free non-bound hormone concentrations can be assessed; (ii) currently clinical reference standards for salivary hormone levels do not exist (as for blood); and (iii) urinary hormone values are influenced by a time-lag effect that is dependent upon glomerular filtration rates [72, 73].

### The Applied Research Environment

The perceived complexity of elite sport training and competition environments is often cited as a justification for low-quality approaches to establishing menstrual cycle phases [74–79]. The numerous and varied demands placed upon elite athletes daily present a challenge to their participation in research projects. Consideration of how to maximise athlete engagement and adherence should form part of a study plan and several approaches can be utilised to mitigate some of the unique challenges research in elite sport presents.

Involving key sport stakeholders (i.e. athletes, coaches, practitioners) in the development of the research question, study design and project delivery will increase the prioritisation of the study within the sport. Co-designed approaches can help solidify engagement, thus increasing the potential for more impactful outcomes [80]. Coaches should be viewed as an integral part of this process, as they are often gatekeepers to athlete participation, yet poor coach engagement is commonly considered a barrier to implementing research in applied settings [81]. Coaches often feel they lack the requisite knowledge about the menstrual cycle to engage in useful conversations with athletes [82], so proactive education strategies, implemented prior to the start of a research project, may prove successful at increasing athlete and coach ‘buy-in’ [83].

Athletes are subject to extensive data collection during the normal course of daily training. It is likely that a transactional relationship between data feedback and adherence exists, which researchers should consider prior to starting a study. Prompt timing, appropriate frequency, and meaningfulness of data feedback will likely promote and maintain athlete engagement [84], as well as helping inform coach and practitioner decision making. Clear feedback pathways should be established, with sensitive data communicated ethically and responsibly.

## Discussion

The increasing volume of research involving female athletes is encouraging. However, the pace of this growth has often outpaced methodological rigour and inclusion of elite-level female athletes. Research findings are frequently incomparable or misleading because of insufficient, inconsistent or inaccurate details. This issue is especially impactful in under-researched and under-resourced populations, as is the case with elite female athletes. This statement provides much-needed recommendations to guide applied high-quality SES studies investigating the effects of ovarian hormone profiles on the health and athletic performance of elite female athletes. Methodological rigour in this area is directly connected to improving research translation rather than adding an unnecessary burden to researchers and participants. To enhance research quality in the least burdensome yet most impactful manner, we propose a reporting checklist, described in Table 3.

**Table 3:** Checklist for reporting studies related to ovarian hormone profiles and [sports]women. Whilst these considerations are provided herein for use in applied studies, the majority of these points are also relevant for basic studies

Needed (topic)	Item no	Rationale
Detailed justification for why ovarian hormone profiles were studied	1	All study rationales,including *novelty*, must be adequately justified, acknowledging if data are being prospectively collected or retrospectively mined to reduce the risk of unsound studies (i.e*.* ill-considered studies). This is especially important in a research area where resources are limited Research on ovarian hormone profiles enhances *equity* (i.e. the genuine need for an investigation in women specifically, to advance their specific development). This is not to be confused with *equality* (i.e. performing the same studies in women as in men for equal representation) that must be equally justified but may not include research on ovarian hormone profiles
Full description **and** justification of which ovarian hormone profiles were studied	2	There are many different ovarian hormone profiles, and it is not likely to be able to include all of them in one study. The choice of included profiles speaks to the potential reach and application of the dataset
Definition of each ovarian hormone profile used, **including** supporting citation	3	Because of the lack of consensus regarding the terminology used to describe different ovarian hormone profiles, a clear definition of the ovarian hormone profile studied is needed, such that a meaningful comparison can be made between studies
Clear statement of whether ovarian hormone profiles were assumed or verified **and** justification of approach taken	4	See Burden et al. [13] and Elliott-Sale et al. [15] for an in-depth commentary on why assumed ovarian hormone profiles should not be used in future studies
Complete description **and** justification of how ovarian hormone profiles were initially classified	5	Given that the verification process is usually conducted retrospectively, and is rarely conducted at recruitment, it is important that readers are clear on how participants were recruited (i.e. identified and selected)
Thorough description **and** justification of how ovarian hormone profiles were verified	6	This information is necessary for readers to establish the quality of the research design. It is important to note that the term ‘research quality’ should reflect the design, execution and reporting of the study. If the design or execution of a study is less than high quality, which is often the case with applied studies, the reporting should clearly and honestly describe any limitations and their potential impact, such that the readers can accurately interpret the data alongside the constraints of the study
Justification of pre-measurement ovarian hormone profile tracking protocols **and** time frame when relevant	7	The processes and time frames associated with pre-measurement ovarian hormone profile tracking should be considered and noted in advance of undertaking the study, to allow for more accurate and precise subsequent measurement timepoints
Justification of number of measurement cycles **and** timepoints	8	A single measurement cycle or timepoint is unlikely to reflect the true situation or outcome given the inter-variability and intra-variability in ovarian hormone profiles. As such, several measurement cycles are recommended
Detailed description of the intended use of the data	9	In applied studies, the intended use of the data needs to be stated, given that applied studies are intended to result in solutions to problems rather than theoretical knowledge
Transparent **and** honest description on any deviations away from the described population and/or research design	10	Any deviations from the intended research design should be reported, such that readers are aware of any breaches to the integrity of the dataset, which could affect the application of the findings
Definition **and** justification of operationalisation and defining of operational terms	11	To aid with the readability, interpretation and application of the study, operational definitions should be provided, especially in this research area, which has [historically] been overlooked in both educational and research settings, meaning that many readers could be new to it. This will also aid with comparisons between studies, which is especially important in an area with limited outputs
Clear statement if validated tools/tests have **or** have not been used	12	The ‘standards/quality’ of the measurements employed should be provided, such that readers are aware of any limitations, and their potential impact, associated with the approach taken
Details on the repeatability of measures employed	13	Given the large inter-individual and intra-individual variation [heterogeneity] in ovarian hormone profiles, it is imperative that the methods employed are capable of discerning any influence of ovarian hormones on the outcomes tested
Access to individual data when relevant	14	Given the large inter-individual and intra-individual variation [heterogeneity] in ovarian hormone profiles and that not all women are affected by their ovarian hormone profiles, individual data should be included, such that readers can access and interpret individual responses to support theory refinement
Objective and subjective measures should be clearly identified and labelled in a visible manner; this includes symptoms (i.e. subjective) vs signs (i.e. objective)	15	It is not the responsibility of the reader to guess if a measure is objective or subjective or the potential impact of objective vs subjective measures on the results. This should be clearly stated to aid with the application and generalisability of the findings
Rationale for statistical approach (including missing data)	16	The chosen statistical approach must be justified in relation to the research question, study design and characteristics of the dataset, with attention to the high inter-individual and intra-individual variability common in ovarian hormone research. A clear rationale ensures that readers can evaluate whether the analysis appropriately reflects the structure and limitations of the data (e.g. repeated measures, clustering, non-normality) Handling of missing data must also be described and justified, as incomplete datasets are common owing to the challenges of repeated hormone profiling. Transparent reporting of whether data were excluded, imputed or modelled as missing is essential
Reflection on the overall quality **and/or** methodological imperfections of the work, based upon published standards	17	Given that those undertaking the research are suitably qualified or experienced to do so, it is the responsibility of the authors/researchers to comment on the overall quality and methodological imperfections of their study using appropriate benchmarking tools/systems/reference points. It is not the responsibility of the reader to guess if the study is high quality and suitable for use in practice (i.e. if there is a high degree of confidence associated with the findings)
Wider dissemination [media, especially social media] strategy	18	Noting that researchers do not have ownership of how others (e.g*.* mainstream media) portray their content, we propose that researchers have a responsibility to present their data in an honest and responsible manner and correct misrepresentation of their data when this has occurred

The importance of methodological recommendations and a reporting checklist is significant. In applied settings, practitioners’ eagerness for performance-enhancing solutions may lead to the implementation of badly designed interventions based on poor-quality evidence. Such interventions, particularly those lacking appropriate control, ecological validity or specificity to the female elite population, are not neutral. While the intended outcome may be “marginal gains”, the potential for unintended negative consequences should not be underestimated. In high-performance environments, even small detriments to health, recovery or adaptation can carry significant costs.

Researchers should adhere to the principle of *primum non nocere*, either help or do no harm. The scientific community must resist the temptation to prioritise speed of publication over robustness of design. Upholding methodological quality is essential for advancing the field and safeguarding athlete health, ensuring that applied recommendations are both meaningful and responsible.

High-quality research outputs require equally high standards of reporting. The adoption of the reporting checklist provides a structured framework to enhance transparency and reproducibility. These should not be perceived as constraints or barriers to research, rather as a shared responsibility of researchers to generate impactful and trustworthy evidence. Such quality assurance measures should be integrated at the study design phase and aligned with the central research question rather than retrospectively applied.

## Limitations of the Statement

This is a rapidly evolving field and although some recommendations may become outdated with advances in techniques and methods, the need for detailed reporting is emphasised. The use of more invasive measurement techniques recommended for accuracy may raise ethical or privacy concerns beyond that described. Additionally, consideration of the breadth and range of cultural, religious, or other intersectional identity characteristics is crucial to reduce stigma and increase inclusivity when discussing or researching ovarian hormone profiles. Finally, the delimitation of the current statement to research in applied elite athlete environments may reduce the generalisability of the statement (and subsequent study findings) to populations with more variability in training, support and lifestyle factors (e.g*.* recreational athletes), where the attributing effects may be more challenging because of additional confounders or colliders.

## Conclusions

While the methodological and logistical challenges of conducting research in elite athletic populations are well recognised, these complexities must not preclude the pursuit of rigorous and externally valid scientific inquiry. A balanced approach is essential; one that prioritises and maintains methodological robustness while accounting for the spectrum of ovarian profiles in elite female athletes. The key recommendations can be summarised as: (1) consider diverse hormone profiles beyond menstrual cycles; (2) use precise terminology and clear definitions of operational terms; (3) formulate precise and unambiguous research questions; (4) align the research questions with appropriate study designs and methods; (5) employ high-quality measurements to establish ovarian hormone profiles; and (6) adopt a reporting checklist to improve research reporting. This presents a promising opportunity to develop credible evidence-based strategies to improve performance, health and well-being in elite female athletes.

## Declarations

### EDI Statement

Our author team consists of 11 female and six male authors: representing senior researchers, clinicians and practitioners from elite international, professional and Olympic sports, from six countries and three continents. The senior corresponding author is female.
